# Supplementary material for: Identification of Freezing-Responsive microRNAs and Their Targets in Chinese Jujube by Small RNA and Degradome Sequencing
Source: Int J Mol Sci. 2026 Sep 4;27(17):7912. doi: 10.3390/ijms27177912 (PMC13566890; doi:10.3390/ijms27177912)
Supplement: Supplementary file 1 [file ijms-27-07912-s001.zip › Supplementary Material S2/novel_miR_17.pdf]

Provisional ID	:	novel_miR_17
Score total	:	0.6
Score for star read(s)	:	-1.3
Score for read counts	:	0
Score for mfe	:	0.3
Score for randfold	:	1.6
Score for cons. seed	:	
Total read count	:	337
Mature read count	:	337
Loop read count	:	0
Star read count	:	0

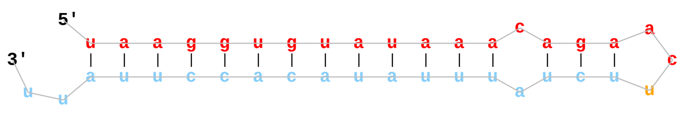

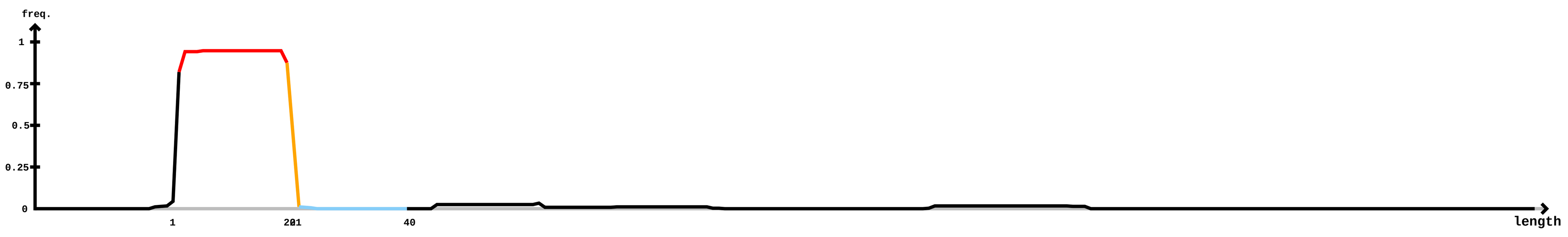

Mature **Star**

```
5'- caaucagcagaagcaaacaaaauaagguguauaaacagaacuucuauuuauacaccuuauuuugguuauucggacccaccaacuaguuacuauaucacauugauagccucuacucauguccuagcucgucugaaagcgagauuuacuucaauuguuugucuauugccuucagcuuuacuuaaguuagcuuccgcuauuucaaaguuuguugagcuuuuuauggaucaaugucacuacccuucucugcau -3'   exp
    ....(((.(((((....(((((((((((((((((.(((...))).)))))))))))))))))(((.....)))....................((((((((.........(((((....((((((.(((((.(((((((..((.......))..)))))..))).)))))....((((.((.((((....)))).))...))))....))))))...)))))))))))))).......))))))))...   reads   mm   sample
    ..................aaaauaagguguauaaacagaa.................................................................................................................................................................................................................   2   0   JCK-3
    ..................aaaauaagguguauaaacagaa.................................................................................................................................................................................................................   1   0   JCK-1
    ..................aaaauaagguguauaaacagaac................................................................................................................................................................................................................   1   0   JCK-3
    ...................aaauaagguguauaaacagaac................................................................................................................................................................................................................   1   0   LT-1
    ....................aauaagguguauaaacagaa.................................................................................................................................................................................................................   1   0   LT-3
    .....................auaagguguauaaacagaac................................................................................................................................................................................................................   4   0   LT-2
    .....................auaagguguauaaacagaac................................................................................................................................................................................................................   1   0   JCK-3
    .....................auaagguguauaaacagaac................................................................................................................................................................................................................   2   0   LT-1
    .....................auaagguguauaaacagaac................................................................................................................................................................................................................   2   0   LT-3
    .....................auaagguguauaaacagaacuu..............................................................................................................................................................................................................   1   0   JCK-2
    ......................uaagguguauaaacagaa.................................................................................................................................................................................................................   3   0   JT-2
    ......................uaagguguauaaacagaa.................................................................................................................................................................................................................   1   0   JCK-1
    ......................uaagguguauaaacagaa.................................................................................................................................................................................................................   1   0   JT-3
    ......................uNagguguauaaacagaa.................................................................................................................................................................................................................   1   1   JT-2
    ......................uaagguguauaaacagaC.................................................................................................................................................................................................................   5   1   LCK-3
    ......................uaagguguauaaacagaa.................................................................................................................................................................................................................   2   0   JCK-2
    ......................uaagguguauaaacagaa.................................................................................................................................................................................................................   7   0   LT-3
    ......................uaagguguauaaacagaa.................................................................................................................................................................................................................   2   0   LT-2
    ......................uaagguguauaaacagaac................................................................................................................................................................................................................   26   0   LT-2
    ......................uaagguguauaaacagaac................................................................................................................................................................................................................   49   0   LCK-3
    ......................uCagguguauaaacagaac................................................................................................................................................................................................................   1   1   LT-1
    ......................Caagguguauaaacagaac................................................................................................................................................................................................................   1   1   JCK-1
    ......................uaagguguauaaacagaac................................................................................................................................................................................................................   17   0   JCK-2
    ......................uaagguguauaaacagaac................................................................................................................................................................................................................   62   0   LT-3
    ......................uaagguguauaaacagaac................................................................................................................................................................................................................   42   0   LT-1
    ......................uaagguguauaaacagaac................................................................................................................................................................................................................   17   0   JCK-1
    ......................uaagguguauaaacagaac................................................................................................................................................................................................................   26   0   JCK-3
    ......................uaagguguauaaacagaac................................................................................................................................................................................................................   14   0   JT-1
    ......................uaagguguauaaacagaacu...............................................................................................................................................................................................................   1   0   LT-1
    ......................uaagguguauaaacagaacA...............................................................................................................................................................................................................   2   1   LT-1
    .......................aagguguauaaacagaac................................................................................................................................................................................................................   8   0   LT-1
    .......................aagguguauaaacagaac................................................................................................................................................................................................................   10   0   LCK-3
    .......................aagguguauaaacagaac................................................................................................................................................................................................................   9   0   LT-2
    .......................aagguguauaaacagaac................................................................................................................................................................................................................   2   0   JCK-3
```

```
                      Mature     Star
caaucagcagaagcaaacaaaauaagguguauaaacagaacuucuauuuauacaccuuauuuugguuauucggacccaccaacuaguuacuauaucacauugauagccucuacucauguccuagcucgucugaaagcgagauuuacuucaauuguuugucuauugccuucagcuuuacuuaaguuagcuuccgcuauuucaaaaguuuguugagcuuuuuauggaucaaugucacuacccuucucugcau
.......................aagguguauaaacagaac.................................................................................................................................................................................................................    6    0    LT-3
.......................aagguguauaaacagaacu................................................................................................................................................................................................................    2    0    LT-1
.......................aagguguauaaacagaacA................................................................................................................................................................................................................    1    1    JCK-1
.......................aagguguauaaacagaacu................................................................................................................................................................................................................    2    0    LT-3
.......................aagguguauaaacagaacA................................................................................................................................................................................................................    1    1    JCK-3
.......................aagguguauaaacagaacA................................................................................................................................................................................................................    2    1    JCK-2
.......................aagguguauaaacagaCcuuc..............................................................................................................................................................................................................    1    1    JCK-2
..........................guguauaaacagaacuucu.............................................................................................................................................................................................................    2    0    LT-1
.................................................................uuauucggacccaccaUc.......................................................................................................................................................................    7    1    JT-3
.................................................................uuauucggacccaccaUc.......................................................................................................................................................................    2    1    JT-2
..................................................................................cuaguuacGauaucacauugauagccucu...........................................................................................................................................    2    1    JCK-2
..................................................................................cuaguuacGauaucacauugauagccucu...........................................................................................................................................    1    1    JCK-3
...............................................................................................cacauugauagccucuac.........................................................................................................................................    1    0    LT-3
...................................................................................................................................................ucaauuguuugucuCuugccuuca...............................................................................    1    1    LT-1
....................................................................................................................................................caauuguuugucuCuugccuucagcu............................................................................    3    1    JT-3
....................................................................................................................................................caauuguuugucuCuugccuucagcu............................................................................    2    1    JT-2
```
